# Complete Genome Sequence of Halomonas sulfidaeris Strain Esulfide1 Isolated from a Metal Sulfide Rock at a Depth of 2,200 Meters, Obtained Using Nanopore Sequencing

**DOI:** 10.1128/MRA.00327-19

**Published:** 2019-06-06

**Authors:** Motofumi Saito, Akane Nishigata, Josephine Galipon, Kazuharu Arakawa

**Affiliations:** a Institute for Advanced Biosciences, Keio University, Tsuruoka, Japan; b Systems Biology Program, Graduate School of Media and Governance, Keio University, Fujisawa, Japan; c Faculty of Environment and Information Studies, Keio University, Fujisawa, Japan; University of Maryland School of Medicine

## Abstract

We report the complete genome sequence of Halomonas sulfidaeris ATCC BAA-803, isolated from a metal sulfide rock at a depth of 2,200 m in the Northeast Pacific Ocean. The assembled genome comprised one circular chromosome of 4.20 Mb and one large plasmid of 273 kb. The chromosome harbors 6,705 protein-coding genes.

## Abstract (precis)

We report the complete genome sequence of Halomonas sulfidaeris ATCC BAA-803, isolated from a metal sulfide rock at a depth of 2,200 m in the Northeast Pacific Ocean. The assembled genome comprised one circular chromosome of 4.20 Mb and one large plasmid of 273 kb.

## ANNOUNCEMENT

Halomonas sulfidaeris strain ATCC BAA-803 (=CECT 5817 = DSM 15722) is a Gram-negative rod-shaped aerobic bacterium classified in the phylum *Proteobacteria*, order *Oceanospirillales*, and family *Halomonadaceae*. It was first isolated from metal sulfide rock at a 2,200-m depth in the Main Endeavor Field of the Endeavor Segment of the Juan de Fuca Ridge, Northeast Pacific Ocean, as strain Esulfide1 (1). It grows optimally at 30°C (pH 7) with 18% total salts. Deep-sea halomonids are of interest for their ability to grow at a wide range of temperatures and potential for seafloor bioremediation. However, very few complete genomes are yet available for these organisms. H. sulfidaeris Esulfide1 is of particular interest due to its proximity to hydrothermal vents and the type of rock from which it was sampled.

The strain was cultured using ATCC medium (1097 *Halomonas* medium, with Casamino Acids replaced by high-molecular-weight casein). The liquid culture was passaged two times overnight at 30°C in a shaker and harvested at an optical density at 600 nm (OD_600_) of ≈0.6. Genomic DNA was extracted using a Genomic-tip 20/G kit (Qiagen). A sequencing library was prepared with a rapid barcoding kit (SQK-RBK004) and was subsequently sequenced using an R9.4 flow cell (FLO-MIN 106) on a GridION X5 system (Oxford Nanopore Technologies).

Adapters were removed, and all 98,820 reads were demultiplexed using Porechop version 0.2.3. The average read length was 4,484 bp, with an *N*_50_ value of 8,468 bp (maximum read length, 109,205 bp) for a coverage of 98×. The reads were assembled using Canu version 1.7.1 (2), and the overlapping end was manually removed to finish circularization. The reads were mapped to the draft 4.20-Mb Nanopore genome using the Burrows-Wheeler Aligner “mem” (BWA-mem) algorithm version 0.7.17 (3). The alignment file was then sorted and indexed with SAMtools (4), and consensus was called using Racon (5). The genome sequence was further polished with Nanopolish version 0.9.2 (6) with minimap v2.5-r622-dirty (7). The quality of the assembly was assessed by calculating the genome completeness using gVolante version 1.2.0 (8), which showed 87.5% BUSCO completeness, indicating a certain amount of uncorrected indels. The genome sequence was annotated by DFAST (9). All software programs were used with default settings.

*H. sulfidaeris* strain Esulfide1 (ATCC BAA-803) has a circular chromosome of 4,207,221 bp and one plasmid with a length of 273,549 bp. The chromosome has a G+C content of 53.8% and contains 6,785 predicted genes encoding 6,705 proteins, 61 tRNAs, and 18 rRNAs. The genome size was similar to that of the contig-level assembly of *H. sulfidaeris* strain SST4, isolated from a trench in South Shetland (NCBI RefSeq accession no. NZ_QNTU00000000). Strain ATCC BAA-803 additionally contained one plasmid that is absent from SST4, with a length of 273 kb and a G+C content of 53.4%. The G+C contents of the chromosome and plasmid were equivalent, and the plasmid shared homology with the plasmid of *Halomonas* sp. strain KO116 (GenBank acccession no. CP011053) and the plasmid of Halomonas ventosae strain NRS2HaP1 (GenBank accession no. CP022738), albeit 40 kb shorter and 155 kb longer, respectively. It contained 412 protein-coding genes, 400 of which were of unknown function (hypothetical protein). Interestingly, out of the 12 coding sequences for which a function could be predicted, 4 were involved in resistance to heavy metals, including mercury, cadmium, and lead. The new plasmid also harbored an IS*256* family transposase that was absent from both CP011053 and CP022738, an indication of active transposable elements (10). The completeness and quality of this genome will allow genome-wide comparison analysis with other species of Halomonas.

### Data availability.

The chromosome and plasmid sequences reported here were deposited in DDBJ/ENA under the accession no. AP019514 and AP019515 and in the Sequence Read Archive (SRA) under BioProject accession no. PRJNA521444.
